# SampPick: Selection of a Cohort of Subjects Matching a Population HLA Distribution

**DOI:** 10.3389/fimmu.2019.02894

**Published:** 2019-12-20

**Authors:** Joseph R. McGill, Osman N. Yogurtcu, Daniela Verthelyi, Hong Yang, Zuben E. Sauna

**Affiliations:** ^1^Hemostasis Branch, Division of Plasma Protein Therapeutics, Office of Tissues and Advanced Therapies, Center for Biologics Evaluation and Research, U.S. Food and Drug Administration, Silver Spring, MD, United States; ^2^Office of Biostatistics & Epidemiology, Center for Biologics Evaluation and Research, U.S. Food and Drug Administration, Silver Spring, MD, United States; ^3^Office of Biotechnology Products, Office of Product Quality, Center for Drugs Evaluation and Research, U.S. Food and Drug Administration, Silver Spring, MD, United States

**Keywords:** immunogenicity, HLA-typing, optimization, donor-selection, simulated-annealing, algorithm

## Abstract

Immune responses to therapeutic proteins and peptides can adversely affect their safety and efficacy; consequently, immunogenicity risk-assessments are part of the development, licensure and clinical use of these products. In most cases the development of anti-drug antibodies is mediated by T cells which requires antigen presentation by Major Histocompatibility Complex Class II (MHCII) molecules (also called Human Leucocyte Antigen, HLA in humans). Immune responses to many protein therapeutics are thus HLA-restricted and it is important that the distribution of HLA variants used in the immunogenicity assessments provides adequate coverage of the target population. Due to biases inherent to the collection of samples in a blood bank or donor pool, simple random sampling will not achieve a truly representative sample of the population of interest. To help select a donor cohort we introduce SampPick, an implementation of simulated annealing which optimizes cohort selection to closely match the frequency distribution of a target population or subpopulation. With inputs of a target background frequency distribution for a population and a set of available, HLA-typed donors, the algorithm will iteratively create a cohort of donors of a user selected size that will closely match the target population rather than a random sample. In addition to optimizing the HLA types of donor cohorts, the software presented can be used to optimize donor cohorts for any other biallelic or monoallelic trait.

## Introduction

Protein and peptide therapeutics include seven of the 10 top-selling drugs (1) and provide medical interventions for diseases that are otherwise untreatable. Immunogenicity, the undesired immune response to a protein or peptide therapeutic, is a key concern during drug-development and licensure. While the development of some drugs has been discontinued due to immunogenicity-risk (2–5); immunogenicity issues continue to cast a shadow even on marketed drugs. For example, within 5 years, 30–70% of patients receiving TNF-alfa inhibitors experience “secondary failure” due to immunogenicity (6). Similarly, about a quarter of hemophilia A patients develop so-called inhibitors, i.e., neutralizing antibodies (NABs) to Factor VIII, leading to severely diminished quality of life and medical costs that can exceed USD 1 million per year. Thus, the most egregious consequence of immunogenicity is not that a drug will fail to be marketed but that medications with a market value of almost 100 billion dollars that treat millions of individuals are sub-optimal.

The immunogenicity risk of a drug-candidate can be determined at two principal steps during drug-development. In early stage drug-development, non-clinical *in silico, in vitro*, and *ex vivo* tools can be used to assess the potential for an immune response (7). Although there have been substantive improvements in these technologies in the last decade, it is still not possible to rely entirely on the surrogate markers measured by these methods for estimating the risk of clinical immunogenicity of biologics (8). Consequently, the identification of anti-drug antibodies (ADAs) and NABs is almost always a part of Phase 3 clinical studies (9).

The HLA-type of a patient is one of several risk factors for immunogenicity. The HLA proteins act at the interface between the antigen and the immune system. These receptors bind peptides derived from protein antigens and transport them to the membrane surface where the complex is recognized by T cells which can then initiate the cascade of complex immune responses. Numerous studies indicate that immune responses to therapeutic proteins require T-cell activation (10). Hence antigen presentation via the HLA is a necessary, albeit not a sufficient, condition for therapeutic protein product immunogenicity (8).

From the point of view of assessing the immunogenicity risk of a protein-drug; a population that has a diverse HLA repertoire presents a challenge. Genes for the major histocompatibility complex (MHC), also called the HLA in humans, are the most polymorphic in the vertebrate genome (11). If, and this is often the case, immune responses to the therapeutic-protein are HLA restricted, ensuring that a representative distribution of HLA variants is included in the clinical and non-clinical studies is very difficult.

A testing cohort can be generated from any “available population” such as HLA typed individuals donating at a blood bank, a bio-repository, commercial catalogs of HLA typed cells etc. The immense diversity of the HLA repertoire raises many technical questions in the design of a study. How many HLA variants should be studied? How does one generate a suitable cohort that considers the relative frequencies of HLA variants in different human populations? For an *ex vivo* assay how many samples should be used? What HLA types should the donors of the cells have? The answers to many of these questions will depend on the drug, the disease and the specific question(s) the study is being designed to answer. However, once a decision has been made as to the composition of the “representative cohort” (e.g., a distribution of HLA alleles reflecting the US population, a disease etc.) statistical approaches can be used to select the most appropriate cohort for the study.

Usual methods for donor cohort selection involve either hand selection of donors to ensure that alleles with high frequencies in the population are included in the study, or random selections of donors under the assumption that this random selection will be a representative sample of the population from which it is drawn. While hand selecting donors to cover important alleles will ensure that these alleles are included in the study, it does not consider the frequencies of the alleles. Additionally, it does not try to model the distribution of the less frequent alleles on the population of interest.

Random selections of donors would address these issues of attaining the proper distribution of alleles assuming the pool of donors is representative of the population from which it is drawn. It is known that some biases will exist in donors in a bio-repository or blood bank (12). In order to confront the biases inherent to the group of samples to choose we propose avoiding random sampling from a biased population and introduce a method that uses simulated annealing to generate a cohort of subjects in which HLA alleles occur at the similar frequencies as they occur in the sub-population of interest. We use a simulated annealing algorithm to select a cohort of subjects that better resembles a background population (vis-à-vis relative frequencies of HLA alleles) as measured by the Jensen-Shannon distance (JSD). While there are several distance measures that can be used, this distance measure is often used in the comparison of probability distributions in machine learning applications due to its symmetry and finite bounds.

Simulated annealing is a global optimization algorithm which draws its inspiration from the metallurgical process of annealing. This annealing process involves a scheduled heating and cooling process that serves to strengthen metal and reduce flaws. The basis of Simulated Annealing is the Metropolis-Hastings Algorithm in which a change is introduced into the system. In our case this change is the substitution of one potential member of the selected cohort for another. This new state is accepted with some probability and the algorithm will continue anew. The introduction of the concept of cooling in annealing sets up a schedule for the acceptance of new states. At the start of the algorithm, the system is hotter and has a great deal more free energy (temperature of 1). This temperature will exponentially decrease over the iterations of the algorithm until the system has cooled to a temperature nearing 0. For each iteration, the JSD score is assessed after the substitution is made. If the substitution is an improvement with respect to JSD, then the change is accepted. In addition, if a random number from a uniform distribution between 0 and 1 is below the current temperature, the change is also accepted regardless of whether the score is an improvement. These non-optimal changes are considerably more likely when there is a lot of free energy in the system (higher temperature). As the system “cools” to a temperature of 0, the chance of a substitution being accepted without also being an improvement in score exponentially decreases. The benefit of this algorithm is in its flexibility in dealing with local vs. global minima. If the algorithm has no chance to accept non-optimal solutions, there is a chance that it will miss a lower minimum and just be stuck in the minimum for the region in which it started. By allowing for the algorithm to try out other areas in the solution space, there is a greater chance that the global minimum is found. Importantly, the reduction in temperature means that toward the later parts of the algorithms run, the algorithm would have hopefully found the area around the global minimum and will fine-tune its selection in that region as the system further cools toward 0.

We demonstrate that a cohort selected using this tool is closer in HLA type distribution to a known background distribution (as measured by the Jensen-Shannon distance) than random selections from biased sets of donors. Finally, we provide illustrative applications of our tool.

## Methods

### Obtaining the Background Distribution of HLA DRB1 Alleles

We obtained the population frequencies of 514 HLA-DRB1 alleles from the United States (US) from the Be the Match® bone marrow donor registry (13). This dataset reports the allele frequencies for broad race categories in the US (Table 1) from 5,745,199 haplotyped samples. To calculate the allele frequencies representing the general American population we weighted the racial allele frequencies with the demographic distribution of those races in the population. To obtain the racial distributions, we first retrieved the US Census Bureau's July 1, 2018 estimates on the population demographics and then amended the percentages such that they add up to a 100, by distributing the missing (100%-99.5%) based on the original percentages. In the allele dataset, there were four ambiguous (04:07G, 11:01G, 12:01G, 14:01G) and four not-expressed (07:10N, 12:24N, 15:17N, 15:50N) DRB1 allele types. For standardization purposes we mapped those alleles to their base allele type (e.g., 04:07G reverts to 04:07). The resulting scaled and weighted table of allele frequencies (Supplementary Table 1) was used as a background distribution for the North American Population.

**Table 1:** The samples used for the creation of the background distribution of HLA-DRB1 alleles for the US population.

**Race/ethnic description**	**DRB1-typed sample counts (1)**	**% US population race distribution (US Census Bureau estimate) (2)**	**% US population race distribution (used in this work)**
African American	505,250	13.4	13.5
Asian or Pacific Islander	568,597	6	6.0
Caucasian	3,912,440	60.7	61.0
Hispanic	712,764	18.1	18.2
Native American	46,148	1.3	1.3
Total	5,745,199	99.5	100

*The allelic distributions for each sub-population are weighted by the demographic distribution of those sub-populations (scaled to 100%)*.

### The Source of HLA Typed Donors

As a test case for an available population of samples, all available samples from the CTL ePBMC® Searchable Database v1.2.6 were used (Table 2). Samples that had no information for HLA-DRB1 were not considered and sample labeled as ambiguous with a “P” group suffix were excluded.

**Table 2:** All available samples from the CTL ePBMCR Searchable Database v1.2.6 were used as an example of available donors to use in the selection process.

**Subject**	**DRB1 Allele 1**	**DRB1 Allele 2**
LP_7	DRB1*07:01	DRB1*14:02
LP_88	DRB1*04:04	DRB1*08:01
LP_89	DRB1*13:02	DRB1*14:01
LP_91	DRB1*04:07	DRB1*07:01
LP_92	DRB1*15:01	DRB1*15:01
LP_96	DRB1*03:01	DRB1*04:04
LP_98	DRB1*03:01	DRB1*03:02
LP_99	DRB1*03:01	DRB1*08:02
LP_105	DRB1*03:01	DRB1*15:01
LP_118	DRB1*14:02	DRB1*15:01
LP_120	DRB1*04:07	DRB1*08:02
LP_121	DRB1*01:01	DRB1*16:02
LP_123	DRB1*07:01	DRB1*13:01
LP_141	DRB1*01:02	DRB1*13:03
LP_151	DRB1*10:01	DRB1*14:02
LP_161	DRB1*03:01	DRB1*14:06
LP_169	DRB1*11:04	DRB1*15:01
LP_178	DRB1*04:02	DRB1*14:06
LP_180	DRB1*01:02	DRB1*03:01
LP_188	DRB1*04:02	DRB1*13:03
LP_194	DRB1*11:04	DRB1*14:06
LP_198	DRB1*04:07	DRB1*16:02
LP_203	DRB1*01:03	DRB1*16:01
LP_205	DRB1*03:01	DRB1*07:01
LP_208	DRB1*03:01	DRB1*11:02
LP_209	DRB1*08:01	DRB1*15:02
LP_210	DRB1*04:10	DRB1*15:01
LP_211	DRB1*01:02	DRB1*04:04
LP_212	DRB1*08:01	DRB1*15:02
LP_214	DRB1*11:04	DRB1*14:06
LP_231	DRB1*11:01	DRB1*11:01
LP_235	DRB1*01:02	DRB1*04:04
LP_238	DRB1*07:01	DRB1*11:04
LP_239	DRB1*07:01	DRB1*08:02
LP_242	DRB1*08:04	DRB1*14:06
LP_253	DRB1*04:04	DRB1*13:03
LP_258	DRB1*07:01	DRB1*14:02
LP_260	DRB1*14:01	DRB1*15:01
LP_263	DRB1*01:01	DRB1*03:01
LP_264	DRB1*04:03	DRB1*08:02
LP_265	DRB1*01:02	DRB1*13:01
LP_266	DRB1*04:03	DRB1*15:01
LP_267	DRB1*04:07	DRB1*16:01
LP_268	DRB1*04:01	DRB1*08:02
LP_270	DRB1*11:01	DRB1*16:01
LP_272	DRB1*08:04	DRB1*15:03
LP_273	DRB1*01:02	DRB1*04:07
LP_275	DRB1*03:01	DRB1*09:01
LP_277	DRB1*01:03	DRB1*01:03
LP_279	DRB1*13:02	DRB1*14:06
LP_280	DRB1*01:01	DRB1*01:01
LP_282	DRB1*04:04	DRB1*12:01
LP_283	DRB1*13:04	DRB1*13:04
LP_284	DRB1*04:07	DRB1*07:01
LP_285	DRB1*03:02	DRB1*07:01
LP_289	DRB1*13:02	DRB1*13:04
LP_290	DRB1*04:01	DRB1*12:01
LP_292	DRB1*14:01	DRB1*15:01
LP_296	DRB1*11:01	DRB1*15:02
LP_297	DRB1*04:04	DRB1*04:07
LP_298	DRB1*04:11	DRB1*16:02
LP_299	DRB1*04:07	DRB1*08:03
LP_301	DRB1*09:01	DRB1*14:02
LP_302	DRB1*14:01	DRB1*15:01
LP_304	DRB1*01:02	DRB1*13:01
LP_305	DRB1*07:01	DRB1*14:01
LP_306	DRB1*11:04	DRB1*13:02
LP_307	DRB1*04:04	DRB1*08:02
LP_311	DRB1*08:02	DRB1*11:01
LP_312	DRB1*14:06	DRB1*15:01
LP_313	DRB1*07:01	DRB1*07:01
LP_314	DRB1*04:02	DRB1*04:02
LP_315	DRB1*13:02	DRB1*13:04
LP_317	DRB1*04:11	DRB1*11:01
LP_318	DRB1*04:04	DRB1*04:04
LP_320	DRB1*01:01	DRB1*16:02
LP_321	DRB1*04:02	DRB1*04:02
LP_323	DRB1*03:01	DRB1*13:02
LP_325	DRB1*12:01	DRB1*13:01
LP_326	DRB1*08:06	DRB1*11:01
LP_327	DRB1*14:02	DRB1*15:03
LP_328	DRB1*01:01	DRB1*11:04
LP_331	DRB1*04:07	DRB1*08:02
LP_332	DRB1*03:01	DRB1*04:03
LP_333	DRB1*04:01	DRB1*07:01
LP_334	DRB1*11:01	DRB1*15:01
LP_335	DRB1*01:02	DRB1*11:02
LP_336	DRB1*01:01	DRB1*04:01
LP_338	DRB1*03:01	DRB1*04:07
LP_340	DRB1*01:01	DRB1*15:01
LP_341	DRB1*01:02	DRB1*13:01
LP_342	DRB1*13:02	DRB1*15:01
LP_343	DRB1*04:01	DRB1*15:03
LP_344	DRB1*04:01	DRB1*07:01
LP_345	DRB1*01:02	DRB1*13:01
LP_346	DRB1*08:01	DRB1*11:01
LP_347	DRB1*13:01	DRB1*13:03
LP_349	DRB1*01:01	DRB1*13:02
LP_350	DRB1*11:04	DRB1*11:04
LP_351	DRB1*11:01	DRB1*13:02
LP_352	DRB1*04:01	DRB1*07:01
LP_353	DRB1*07:01	DRB1*11:01
LP_354	DRB1*07:01	DRB1*07:01
LP_355	DRB1*01:01	DRB1*13:02
LP_356	DRB1*04:07	DRB1*16:01
LP_359	DRB1*04:01	DRB1*07:01
LP_360	DRB1*01:03	DRB1*03:01
LP_361	DRB1*04:04	DRB1*13:01
LP_362	DRB1*08:04	DRB1*11:01
LP_363	DRB1*01:01	DRB1*11:04
LP_364	DRB1*09:01	DRB1*15:01
LP_365	DRB1*04:05	DRB1*08:02
LP_366	DRB1*03:01	DRB1*13:01
LP_367	DRB1*11:02	DRB1*13:02
LP_369	DRB1*01:01	DRB1*11:04
LP_372	DRB1*13:01	DRB1*13:02
LP_373	DRB1*03:01	DRB1*08:02
LP_375	DRB1*03:01	DRB1*04:02
LP_377	DRB1*01:01	DRB1*11:04
LP_378	DRB1*13:03	DRB1*16:01
LP_379	DRB1*04:04	DRB1*07:01
LP_381	DRB1*04:05	DRB1*12:02
LP_383	DRB1*01:02	DRB1*13:01
LP_384	DRB1*09:01	DRB1*13:02
LP_385	DRB1*10:01	DRB1*14:06
LP_386	DRB1*03:01	DRB1*04:11
LP_388	DRB1*03:01	DRB1*03:01
LP_389	DRB1*07:01	DRB1*07:01
LP_390	DRB1*01:01	DRB1*13:02
LP_391	DRB1*04:11	DRB1*13:02
LP_392	DRB1*01:01	DRB1*04:01
LP_393	DRB1*03:01	DRB1*09:01
LP_394	DRB1*03:01	DRB1*14:02
LP_395	DRB1*01:02	DRB1*15:03
LP_396	DRB1*04:05	DRB1*15:01
LP_397	DRB1*04:06	DRB1*15:01
LP_398	DRB1*01:02	DRB1*03:02
LP_399	DRB1*04:01	DRB1*15:01
LP_400	DRB1*01:02	DRB1*12:01
LP_401	DRB1*01:02	DRB1*11:02
LP_402	DRB1*09:01	DRB1*15:01
LP_403	DRB1*04:04	DRB1*07:01
LP_404	DRB1*07:01	DRB1*11:04
LP_405	DRB1*11:01	DRB1*13:04
LP_406	DRB1*07:01	DRB1*11:01
LP_407	DRB1*11:02	DRB1*13:02
LP_408	DRB1*03:01	DRB1*16:01
LP_409	DRB1*13:02	DRB1*16:02
LP_411	DRB1*07:01	DRB1*15:01
LP_412	DRB1*08:02	DRB1*16:02
LP_413	DRB1*04:02	DRB1*07:01
LP_414	DRB1*01:03	DRB1*03:01
LP_415	DRB1*11:01	DRB1*15:02
LP_416	DRB1*03:02	DRB1*08:02
LP_417	DRB1*08:03	DRB1*12:02
LP_418	DRB1*09:01	DRB1*14:02
LP_419	DRB1*10:01	DRB1*11:01
LP_420	DRB1*13:01	DRB1*14:01
LP_421	DRB1*04:07	DRB1*15:01

*Each sample is identified by a unique donor ID and has two HLA-DRB1 alleles identified*.

### The Jensen-Shannon Distance

The Jensen-Shannon Distance is used as a metric for scoring the difference between the two probability distributions. This metric was used since it is symmetrical version of the Kullback-Leibler Divergence. As we are using the natural log in the Kullback-Leibler Divergence definition, the Jensen-Shannon Distance is bounded by 0 and $\sqrt{\ln\left(2\right)}\approx 0.83$. It is defined by the following equation:

$$\begin{array}{l} JSD\left(P||Q\right)=\sqrt{\lambda *D_{KL}\left(P||M\right)+\lambda *D_{KL}\left(Q||M\right)} \end{array}$$

where:

D(P||Q) is the Kullback-Leibler divergence of probability distributions a and b.
λ is a scaling factor for the two distribution (1/2 is generally used).
P is the probability distribution of the Sample of interest.
Q is the probability distribution of the Target Population (Background Distribution).
M is the mean of both distributions defined as:

$$\begin{array}{l} M=\frac{1}{2}\left(P+Q\right) \end{array}$$

The Kullback-Leibler Divergence is defined as:

$$\begin{array}{l} D_{KL}\left(P||Q\right)=\underset{a\varepsilon A}{\sum }P\left(a\right)*\log\left(\frac{P\left(a\right)}{Q\left(a\right)}\right) \end{array}$$

where:

A is the union of all alleles in the target and sample populations.

### Simulated Annealing

**Figure 1:**
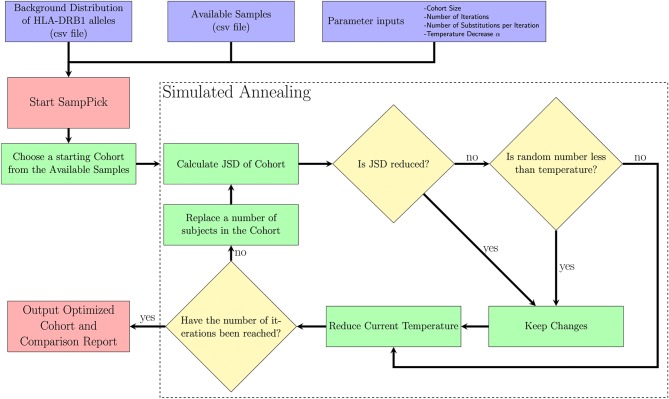
A description of the SampPick algorithm. The user inputs a background distribution, a set of available samples and/or a sample to test, and parameters for the algorithm. The algorithm will proceed for the prescribed number of iterations, making substitutions to the samples chosen in order to obtain the sample closest to the background distribution ranked by Jensen-Shannon Distance.

A Simulated Annealing algorithm (Figure 1) was used to optimize selection of a sample cohort through iterative resampling of the population. The algorithm allows for the user to select a sample size (N), as well as refine the parameters: number of iterations (I), temperature decrease (α), number of changes (C).

The purpose of this algorithm is to accept changes to the selected cohort which will decrease the JSD between the cohort chosen and target distribution. The reason it will accept non-optimal changes in step VII (ii) is to allow the algorithm to avoid getting stuck on local minima and have a chance to fully explore the search space. It should be noted that the probability of accepting non-optimal changes decreases exponentially over time.

- The starting temperature is set to 1.
- A random sample of size N is first selected by the algorithm.
- This sample is scored using the Jensen-Shannon Distance (JSD) described above.
- C members of the sample are replaced with new subjects from the available samples.
- The subjects removed from the sample are put back into the available samples.
- This sample is scored using the JSD.
- This new sample will be accepted if either of two criteria are true:
  - The new sample has a lower JSD score.
  - A number drawn from a uniform distribution, U (0,1), is less than the current temperature.
- The current temperature is multiplied by (1-α).
- Return to step 4 while the number of iterations is below the target number of iterations.
- Output an optimized sample to the user.

The algorithm is written in Python (14). All visualizations were created in R Core Team (15) using the ggplot2 package (16) or in Python using matplotlib (17).

### Parameter Testing

The algorithm was run using the background population of interest and available sample mentioned above. Sample sizes of 10, 20, 30, 40, 50, 75, and 100 were chosen from the available sample of 159 subjects. The number of substitutions during each iteration was all discrete values from 1 to 10. The number of iterations test were: 10^2^, 10^3^, 10^4^,10^5^, and 10^6^ with corresponding values for α of: 0.2, 0.02, 0.002, 0.0001, and 0.00001. These values of α were chosen to allow for the exponential decay of the temperature over the respective number of iterations to approach 0 with sufficient speed.

It should be noted that parameters are highly sensitive to the inputs given and it is often best to try a number of combinations of parameters (as seen in section Optimizing Number of Iterations, Size of Replacement Sub-set, and Cohort Size for Simulated Annealing) to get the best results.

## Results

### Workflow and Description of Computation Tool

We have developed a computational tool that uses a bio-repository of HLA typed cells, and a background distribution of allelic frequencies in the global population or a sub-population of choice. In this study we used an on-line catalog of HLA typed frozen cells, ePBMC® to evaluate the algorithm. The number of unique donors in the catalog fluctuates; at the time of our analysis there were cells from 159 unique donors listed who had non-ambiguous HLA-DRB1 typing. The workflow of the computational tool is illustrated in Figure 1 and described in the section Methods. In our testing of the software, we first developed trials to evaluate the parameters being used in the software. We then present instructive examples that show a variety of uses for this software.

### Optimizing Number of Iterations, Size of Replacement Sub-set, and Cohort Size for Simulated Annealing

As illustrated in Figure 1, our computational tool selects a cohort from the available pool of donors. The input parameters for simulated annealing are an important factor in finding the most compact, yet representative group of donors to use for an experiment. Our algorithm was tested using different numbers of iterations (100, 1000, 10000, 100000), various numbers of substitutions made per iteration (from 1 through 10) and different cohort sizes from 2 to 157.

**Figure 2:**
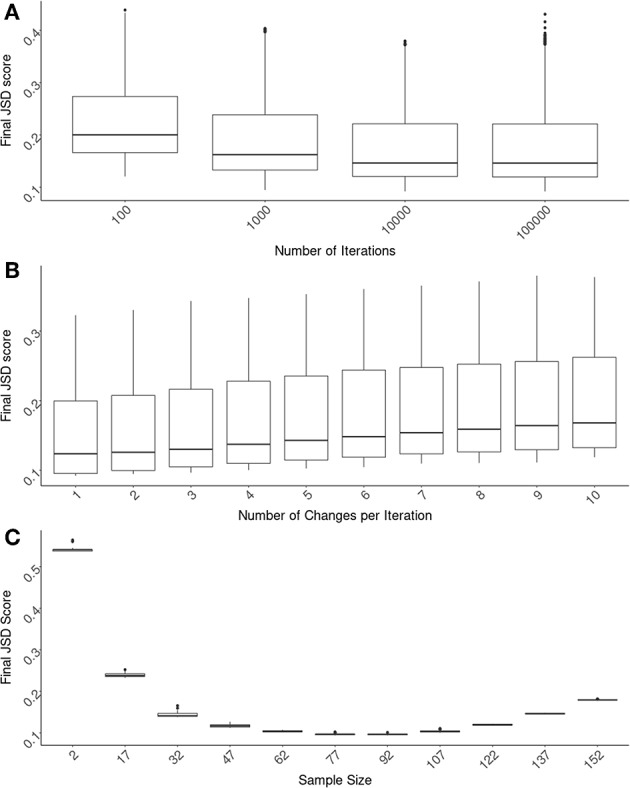
Optimizing SampPick's input parameters. The JSD score was calculated for 100 repetitions for each combination of: a sample size from 2–157, a number of iterations from 100 to 100,000, and a number of substitutions per iteration from 1 to 10. **(A)** The number of iterations for all test were compared and there is a clear decrease of scores as iterations increase. The decrease in JSD scores appears to diminish at 10,000 iterations. **(B)** Looking only at the tests with 10,000 iterations, the number of substitutions per iteration was examined. Simply making one substitution per iteration returned the lowest scores. **(C)** Looking at the tests with 10,000 iterations and 1 substitution per iteration, the JSD scores decrease as sample size increases. At a sample size of 82, the JSD score increases as sample size increases.

The first parameter we evaluated is the number of iterations the algorithm runs for. The JSD scores decrease as one allows the algorithm to run longer. These extra iterations allow for more fine-tuning of the cohort selection. However, there is a limit to the usefulness of this fine-tuning and unnecessary iterations increase in computational time (and costs). We found that 10,000 iterations are optimal (Figure 2A) and used this number for evaluating the other parameters. It is important to note that the α parameter is directly related to the number of iterations chosen. An α needs to be chosen for each number of iterations such that (1 − α)^*i*^ approaches 0 as i approaches the number of iterations. The values we used, as shown in section 2.5 could be used as a guideline for how quickly (1 − α)^*i*^ approaches 0.

Using test runs that all had 10,000 iterations, we evaluated the number of changes allowed per iteration. In these tests we found that the lowest JSD scores were obtained when there was only one change made per iteration (Figure 2B).

The size of the cohort chosen is related to considerations outside of the scope of this algorithm including cost, samples availability, etc. We have assessed several different sample sizes using these original parameters of 10,000 iterations with an α of 0.002 and one change per iteration (Figure 2C). The variation in JSD scores in relation to sample size will be helpful for the user in deciding on an appropriate sample size.

### Performance of the Computational Method and the Range of JSD Scores

**Figure 3:**
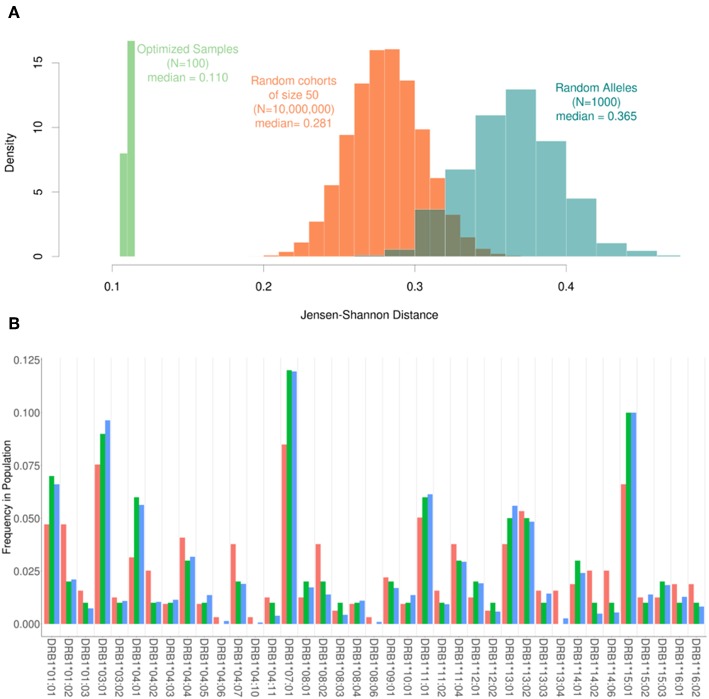
The range of Jensen-Shannon distances. **(A)** 10,000,000 random samples of 50 subjects were drawn from our pool of donors and their similarity to the background distribution of North Americans was calculated using JSD (Orange) with a median value of 0.281. Additionally, 100 samples were created using our optimization algorithm (Green). These 100 samples all had a sample size of 50 and were run using one substitution per iteration and 10,000 iterations. The scores obtained using the algorithm were roughly 7 standard deviations below the mean of the scores for the random samples with a median score of 0.110. In addition, 1000 sets of completely random alleles were selected (Blue). These random sets, which were meant as a negative control, have a median score of 0.365. **(B)** The frequency of alleles in the background distribution of alleles in the North American Population (blue) was compared to both the entire set of 159 subjects available (red) and a smaller subset of 50 subjects selected by the algorithm (green). Although the optimized sample is much smaller than the available samples, it is much closer to the desired distribution of alleles.

Based on the results shown in Figure 2, the following parameters were selected for evaluating the performance of our algorithm: Cohort size (N) = 50; Replacement number per iteration (C) = 1, number of iterations (I) = 10,000. Using our pool of 159 subjects we randomly selected 10-million cohorts of 50 subjects each. The JSD scores for these (compared to the frequencies of HLA variants in the US population) are depicted in the histogram in Figure 3. We also used the same pool of subjects to run our algorithm to select 100 cohorts (of 50 subjects each). The mean of the JSD scores for the cohorts selected by our algorithm (0.11) was 7 standard deviations below the mean JDS scores for the random samples. In addition, 1,000 random sets of 100 alleles, drawn with replacement from the 30 most common in the North American population, were generated and scored according to JSD. These allele sets had the same number of alleles as a cohort of 50 donors, but the alleles were uniformly distributed with respect to HLA type. These sub-sets were used as a negative control to examine the scores of cohorts that were not drawn from the given population distribution at all. These cohorts of random alleles were found to have a higher JSD score than the random donor cohorts, 0.365. This progression of shows that while the random cohorts drawn from actual donors are more representative of the population of interest, the optimized samples that result from our algorithm are far better.

The frequencies of HLA alleles in the pool of 159 subjects compared to the frequencies of the same alleles in the US population (Figure 3A) illustrate considerable discrepancies. A much smaller cohort of 50 subjects drawn from this pool using our algorithm however shows a far closer match to the frequencies of HLA alleles in the population (Figure 3B).

### The Diminishing Returns of Increasing Sample Size

One important utility of the SampPick algorithm is that not only does it choose samples that more closely match the population of interest, but it will often choose fewer samples than available in the pool. Figure 2C shows the JSD scores from 25 runs of the algorithm with sample sizes ranging from 2 to 157 (out of 159 available samples in the pool). It is not surprising that the JSD scores decrease as sample size increases from 2 to ~80. What is crucial for the setup of experiments using a limited donor pool such as this is that at a certain point the JSD score will start to increase, tending toward the sub-optimal score of the whole donor pool. Similar to the constraints imposed on a cohort of extremely small sizes, where it is not possible to generate a representative cohort with 2 to 7 subjects, larger cohorts with 152 or 157 samples are also constrained. In these almost complete sub-sets of the whole donor pool, the algorithm is only able to exclude small numbers of donors to match the population of interest. Our approach therefore not only results in cohorts more representative of the population but also (by limiting the size of the cohort) saves cost, labor, and time.

### Is There a Need for Our Computational Method?

#### Assessing the Distribution of a Donor Cohort for an *ex vivo* Study of Inhibitor Development to Factor VIIa (FVIIa) Analogs Created to Reduce Immunogenicity

**Figure 4:**
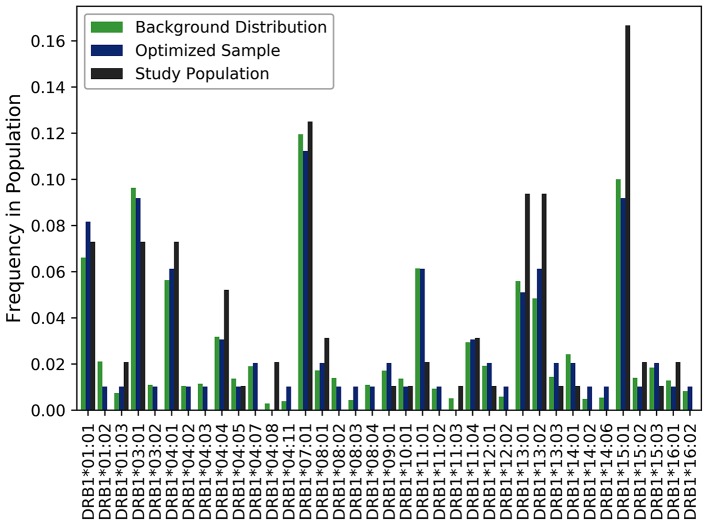
Optimizing selection of a hand-picked donor cohort. A cohort of 50 HLA-typed donors were used in a T-cell proliferation and ELISA assay to assess the T-cell responses of three variants engineered to reduce the immunogenicity of a FVIIa. The frequency of each allele found in the assay is shown in black. The frequency of each allele in the optimized sample is shown in blue. The frequency of each allele in the background distribution of alleles in the North American population is shown in green. The use of our algorithm resulted in a reduction of JSD from 0.198244 in the hand-picked sample to 0.111660 in the optimized sample when each was compared to the background distribution of HLA variants in the US population.

A cohort of 50 HLA-typed donors were used in T-cell proliferation and ELISA assays to assess the T-cell responses of three variants engineered to reduce the potential immunogenicity of FVIIa (18). In this experiment, donors were manually chosen with the intention of being representative of the North American allelic distribution. As shown in Figure 4, the optimized sample shows a closer match to the North American allelic distribution than the samples selected by hand. In addition to avoiding overrepresenting common alleles such as HLA-DRB1^*^15:01, HLA-DRB1^*^13:02 and HLA-DRB1^*^13:02, the algorithm selects alleles such as HLA-DRB1^*^04:02 and HLA-DRB1^*^04:03. The use of our algorithm caused a reduction of JSD from 0.198244 in the hand-picked sample to 0.111660 in the optimized sample.

#### Assessing a Cohort Used in a Clinical Study for Association of HLAs With Neutralizing Antibodies to Factor VIII (FVIII)

**Figure 5:**
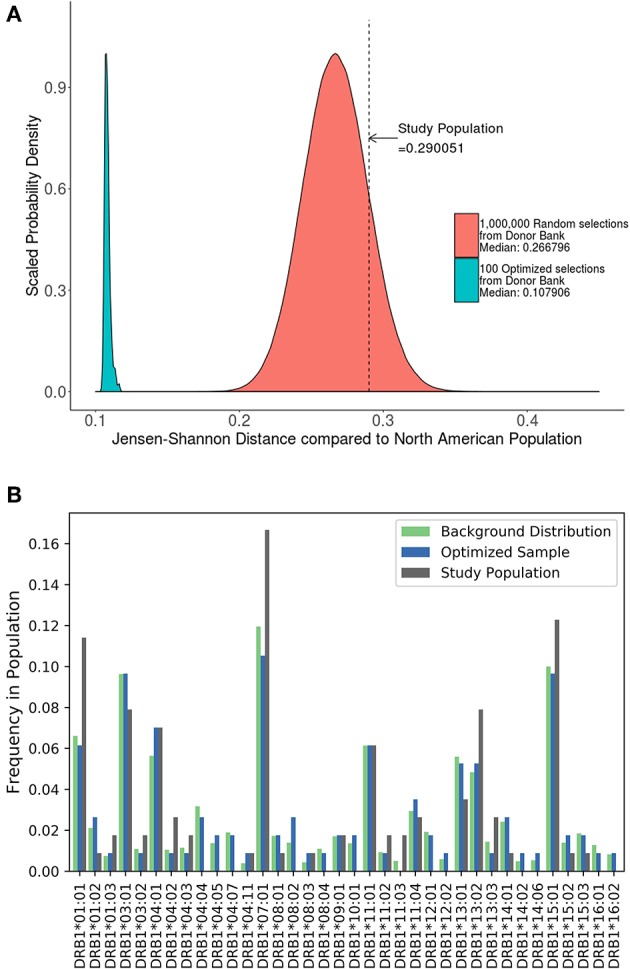
Optimizing a cohort with donors from the same sub-population as the population of interest. **(A)** A cohort of 57 HLA typed subjects forming the control group in a clinical study were analyzed using the algorithm. The study population had a JSD score which fit within the normal bounds for a population size of randomly selected donors from our available samples (red). However, 100 samples of the same size (green) were created from the same available samples using the algorithm. These optimized samples had a significantly lower JSD score. **(B)** An analysis of one of these optimized samples (blue) show that it is much closer in distribution to North American Population (green) than study sample (gray).

A cohort of 57 HLA typed subjects with Hemophilia A was used in a study of neutralizing antidrug antibodies to FVIII (19). As the study was conducted in the US, we estimated the JSD score (0.29) between this cohort and the North American population with respect to HLA-DRB1 allele frequency (Figure 5A). We also generated 1,000,000 random samples of 57 subjects each from the ePBMC samples to obtain a distribution of JSD scores from this population. Since the population in the study and the source of the ePBMC samples is similar (North American), it is not surprising that the study population fit within this distribution. We then ran our algorithm 100 times to create optimized samples from the same pool of ePBMCs. These optimized samples have a much lower JSD score when compared to the allele frequencies in the North American population. In addition to showing a global decrease in the JSD score (Table 3), we also demonstrate that compared to the study cohort our optimized cohort better matches the frequencies of individual HLA variants in the North American population (Figure 5B). Our analysis illustrates that the distribution of HLA alleles in a typical clinical study is sub-optimal if the aim is to have a cohort that is representative of the general population.

**Table 3:** A summary of studies that were analyzed in the testing of SampPick.

**Study**	**Size of background distribution**	**Sample size in study**	**Jensen Shannon distance score of study population**	**Jensen Shannon distance score of optimized cohort selection**
FVII (18)	5,745,199 (13)	50	0.198	0.112
FVIII (19)	5,745,199 (13)	57	0.290	0.108
Psoriasis (20)	70890 (13)	90	0.603	0.168
RA Cases (21)	4280 (22)	744	0.240	N/A^*^
RA Controls (21)	4280 (22)	620	0.129	N/A^*^

*Included are the size of the population used to create the sub-populations background distribution of alleles, the cohort size analyzed, and the JSD score of both the cohort analyzed and the optimized cohort*.

* *These examples do not include optimized samples. They are examples of post-hoc analysis of two study samples*.

#### Comparing the Distribution of HLA Variants in a Biased Cohort, Randomly Selected Individuals, and a Cohort Optimized Using SampPick

**Figure 6:**
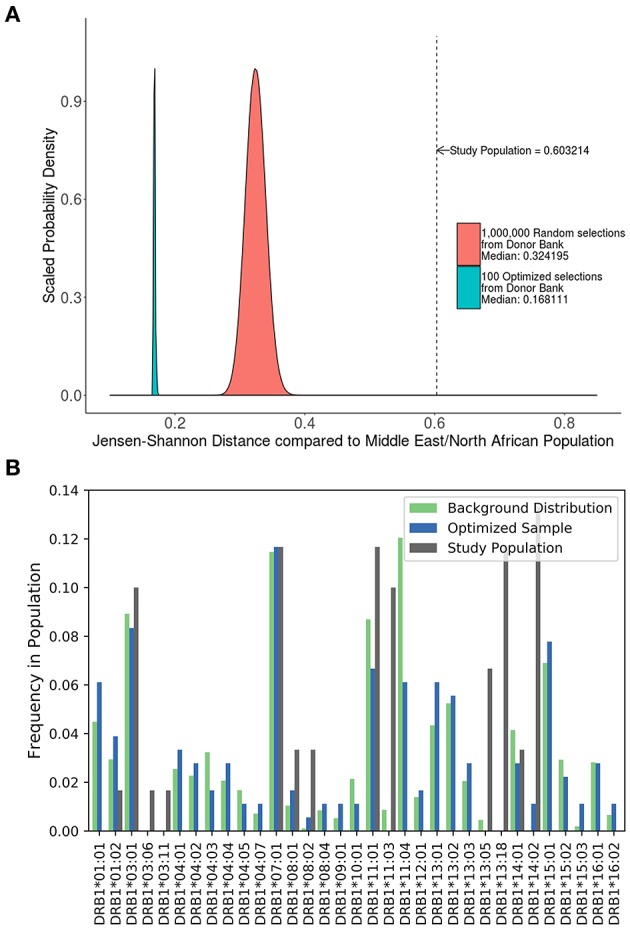
Optimizing a cohort with donors from a different sub-population as the population of interest. **(A)** A similar analysis was performed on the 90 HLA typed subjects who formed the control group of a study on the relationship between HLA-DRB1 type and psoriasis in Iraq. The control population being analyzed was not randomly selected and as such is very different from a background distribution of Middle Easterners/North Africans Donors in the United States. In addition, 1,000,000 million randomly selected samples from a North American cohort of were selected and compared to this same background distribution (red). One hundred optimized samples created from these available North American samples (green) were much closer to the target Middle Eastern/North African population even though they were chosen from a group of general North American Donors. **(B)** The distribution of alleles one of the optimized sample (blue) is much closer to the target background distribution (green) than the control group in the study (gray).

A study carried out in Iraq (20) recruited self-selecting individuals from a hospital and not a randomly selected cohort. This cohort has a much higher JSD score than the distribution of JSD scores of 1,000,000 randomly selected 48 individual samples from the ePBMC® databank as compared to a background distribution of Middle Eastern and North African donors due to the inclusion of some very rare alleles (i.e., DRB1^*^14:02) (Figure 6A). An optimized cohort of 48 subjects had a lower JSD score when compared to either the randomly selected subjects or the subjects included in the study (Figure 6A). This analysis provides another example where study subjects are sub-optimal with respect to distribution of HLA variants (Figure 6B).

#### Evaluating the Distribution of HLA Variants in Cohort of Subjects With Rheumatoid Arthritis (RA) Using SampPick

**Figure 7:**
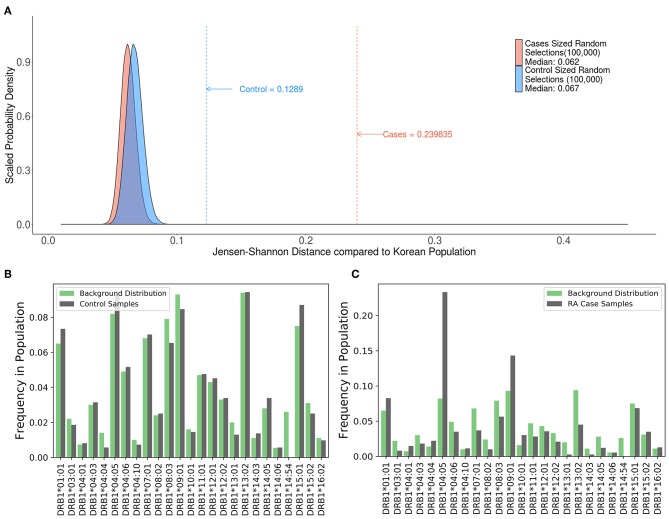
Comparing case and control samples to a sub-population without using optimization. **(A)** An analysis of the relationship between HLA-DRB1 alleles and rheumatoid arthritis (RA) cases in Korean patients show another use for this program. Similarly, sized samples were made for both the 744 RA cases (red) and 620 controls (blue) based on the distribution of HLA-DRB1 Alleles in the Korean population. These samples were created by drawing random alleles from the background population weighted by the calculated frequencies and are expected to be very similar to the background distribution. These samples still have some distance from the background distribution. (median scores of 0.062 and 0.067, respectively). The control (blue dashed line) is much closer to the background distribution than the cases (red dashed line) indicating that the control group is a better match to the background distribution. **(B,C)** While **(A)** shows that there is a difference between the cases and control groups, output from SampPick show that the frequency distribution of the control group (**B**, green) match up with the background distribution of alleles in the Korean population (**B**, gray). The over-representation of certain alleles in the cases group (**C**, green), specifically DRB1*04:05 and DRB1*09:01 is easily identifiable in this graph.

A study by Lee et al. (21) evaluated 1364 HLA typed Korean subjects, 744 patients with RA and 620 healthy controls. Consistent with previous findings, this study demonstrated an association between some HLA haplotypes and RA. We computed the JSD scores for the HLA alleles in the patient population and control subjects as well as a distribution of theoretical samples drawn from the pool of alleles in the population, weighted by allele frequencies found on the Allele Frequencies Net Database (22, 23) (Figure 7A). While the large sample sizes of these theoretical groups coupled with the fact that they were drawn using the theoretical frequencies of the background distribution should render a nearly perfect match, the results show that they still have some distance from the background distribution, with median scores of 0.062 and 0.067. The distribution of HLA alleles in both populations deviated from the distribution of alleles in the Korean population (JSD scores of 0.1289 and 0.2398 for the control and RA populations, respectively) (Table 3). This finding is consistent with our findings in Figures 4–6 and shows that the relative frequencies of HLA variants in randomly picked individuals, deviates from that found in the population. This bias could be due to some bias in either the sample selected or in the background distribution. However, the difference between the JSD scores for the control and patient populations suggests that specific alleles are represented in the patient population. Figures 7B,C show that the differences in allelic distribution is due to large discrepancies in certain HLA-DRB1 alleles in the RA cases as compared to the Korean population that are not exhibited in the controls.

## Discussion

In the last decade novel and critical medical interventions have revolutionized the treatment of many devastating diseases. Their immunogenic potential poses a critical safety and efficacy threat. Therefore, immunogenicity needs to be considered at every step of drug-development and licensure (8). For most biologics, immunogenicity risk is evaluated thought clinical trials, but the size of the trial (and donors for *ex vivo* non-clinical immunogenicity assessments) can range from thousands of patients to <100, depending on the disease. A critical unresolved concern when setting up clinical trials is that adequate and representative HLA distribution is achieved so that the results can be extrapolated to the affected population. Multiple studies have shown that HLA alleles play a central role in the immunological cascade and some immune responses are HLA-restricted (24). The HLA repertoire is the most diverse in the human genome and different HLA alleles occur at different frequencies in different human sub-populations (23). This is illustrated by the difference in relative frequencies of HLA alleles between human ethnicities (25, 26) disease conditions (27, 28), etc. Currently, given the difficulties of putting together a cohort that is representative of the HLA repertoire, most studies do not even collect information on the HLAs represented in the cohort. As shown above, based on a JSD-score (see methods and results) the HLA distribution of study subjects in some studies where HLA typing was performed (Figures 4–7) was not representative of the population from which the population was drawn. This is expected as we find that the HLA distributions of as many as 700 randomly selected individuals deviates from the distribution of HLA variants in the same population to some degree (Figure 7A), but larger trials are expensive, and can delay the access of patients to drugs.

One proposed solution to the seemingly intractable problem of generating a reasonably-sized cohort that includes HLA variants at frequencies comparable to any desired population is to use simulated annealing (29). As previously discussed, self-section bias and the “healthy donor effect” (12, 30) will cause for some bias in the available samples in a blood bank. If a cohort of donors is selected from these biased samples, the HLA allele distribution will closely match unknown, biased population of blood donors rather than the target population. To counteract this, samples can be compared to a known distribution. We assume that the actual distribution of HLA alleles in a population is known or can be estimated using larger samples such as the Be the Match® bone marrow donor registry (13). By optimizing cohort selection to a known distribution, rather than randomly sampling a biased sub-population, the selected cohort of subjects will be a better match of HLA type distribution.

For instance, we demonstrate an improvement in the JSD-score for cohorts of 50 subjects selected using SampPick that is 7 standard deviations below the mean score of 10-million randomly selected cohorts of 50 each (Figure 3A). Importantly, the cohorts of 50 generated using SampPick were constructed from a pool of only 159 HLA typed donors. This indicates that this tool can improve the HLA distribution efficiency of subject selection even in the absence of large numbers of available subjects.

A second advantage of the SampPick algorithm is the ability to generate cohorts based on background distributions of any type. As is seen in the case of RA patients in Korea, the distribution of allele is different than the distribution in healthy donors (Figures 7B,C). By using the frequencies in this background distribution, one could use the algorithm to select a donor cohort to more closely match Korean RA patients. In this case, a chosen population would more closely match the population of people who are receiving a certain treatment.

A third advantage of the SampPick algorithm is the ability to make donor cohorts the match a given subpopulation while drawing from any donor pool. Two insights are gained from the analysis of the cohort of patients from in Iraq (20) (section Is There a Need for Our Computational Method?). First, the scores of randomly chosen samples from a catalog of North American donors is higher when compared to a background distribution of alleles in a population of Middle Eastern/North African donors, medians of 0.32 vs. 0.27 (Table 3); second, optimized samples selected from North American donors, compared to a Middle Eastern/North African background distribution, have a higher score than optimized samples selected from North American donors compared to the North American background distribution. These results should be obvious as drawing a sample from one distribution that would match a completely different distribution by chance while possible; is extremely unlikely. What is notable is that the samples from the catalog of North American donors, optimized to match the Middle Eastern/North African allelic frequencies outperformed random selections of North American donors compared to the North American allelic frequencies. This exhibits the flexibility in the allelic distribution of donor pools while selecting a cohort matching a given sub-population's allelic distribution.

In clinical studies it may often be impractical to use HLA typing as an acceptance or rejection criterion. In addition, specific HLAs may be more frequent in certain disease populations. Further, the risk of developing NABs varies for different therapeutics. This makes it difficult to justify selection of subjects based on their HLA. Nonetheless, as HLA typing becomes more accessible, there is value in evaluating the HLA distribution in the trial cohorts and any deviation between the study population from the population that will be treated. This information can contribute to the totality of the evidence used to evaluate the immunogenicity risk of a therapeutic or a class of products, which includes experience with similar proteins, presence of a comparable endogenous protein, results from non-clinical *ex vivo* studies, etc. Post-marketing, patients with HLA alleles not included in the clinical study but adjudged high-risk based on secondary non-clinical evidence could be monitored more carefully, bringing us closer to personalized medicine.

Another potential application of SampPick is during the development of generic peptides. Peptides smaller than 40 amino acids are considered small molecule drugs and not biologics. In the US, once regulatory protection expires, it may be possible to develop generic copies of synthetic peptides, however; concern about their immunogenicity potential can preclude their licensing in the absence of clinical trials. In some instances, when the immunogenicity of the product is well-understood, bio-analytical studies can provide sufficient information to establish that the generic versions do not pose an increased immunogenicity risk in the absence of clinical trials (5). Such approaches require multiple bioanalytical, *in silico*, and *in vitro* immunological assessments and commonly include assays (e.g., ELISpot assays, T-cell proliferation assays and DC-T assays) that use human PBMC (10). To ensure that these methods provide a meaningful assessment of clinical risk it would be advantageous to use cells from a cohort of donors that is representative of the population that will receive the drug. Such a cohort can be aided by using SampPick to select the donors. Catalogs of banked HLA typed blood and PBMC samples are readily available and we have demonstrated that it is possible to use one such catalog with a listing of 159 donors to generate a cohort of 50 with HLA frequencies comparable to those observed in the US population (Figure 4). Finally, it is increasingly recognized that some HLAs are associated with specific diseases (28) and can have an impact on the responses to medications (31, 32). In studies designed to identify or validate such associations SampPick could prove to be a useful tool.

One potential drawback to SampPick is that it is used on a single feature in our test cases, HLA-DRB1. This is not a problem when working under the assumption that there is a genotype that is the main driver of a systemic response; however, this could be an issue when using this method in other situations. For instance, it has been demonstrated (33) that for peptides derived from the Dengue Virus the overall magnitude of CD4^+^ T cell responses is higher in HLA-DRB1 compared to other HLA class II alleles. However, when antigens (proteins) encoded by the Dengue polyprotein were tested, for some of the proteins the magnitude of CD4^+^ T cell response was higher for HLA-DQ or HLA-DP. Thus, in designing experiments for cases where alleles other than HLA-DRB1 are of interest our software allows addition of a functionality for the creation of individual features describing combinations of multiple alleles. This extension of the software is described in the documentation and enables the users to optimize populations for other biallelic traits, haplotypes or phenotypic traits which may find applications in pharmacogenomic studies. It is worth noting that the increasingly smaller joint frequencies of combinations of alleles or traits will most likely require larger sample sizes to ensure adequate coverage of genotypic combinations.

Additionally, the efficacy of this algorithm is dependent on the sample from which to draw from. While we have shown that cohorts with HLA distributions representing racially different populations can be created using the same pool of donors, it is preferable to use a pool of donors that is similar to the desired population. This is shown in the comparison of the two examples of sample optimization (Figures 6A, 7A). A sample of North American donors was used to generate two cohorts: (i) Matching the HLA distribution of the North American population and (ii) matching the HLA distribution of the Middle Eastern/North African population. We obtained lower JSD scores for the former compared to the latter (0.108 vs. 0.168).

In summary, we have described the development of a computational tool, SampPick, that can be used to assess the distribution of HLA frequencies in cohort of subjects as well as to generate a cohort that is closely matched vis-à-vis HLA frequencies to a target population. We have also provided several examples showing that SampPick could prove useful during the selection of patients or blood donors to improve the development of protein therapeutics, therapies that target the immune system and in clinical studies that evaluate these products. The use of this tool can facilitate the translation of results from *ex-vivo* studies and clinical trials to the patient population.

## Data Availability Statement

All datasets generated for this study are included in the article/Supplementary Material.

## Author Contributions

JM and OY conceptualized and designed the study under the supervision of HY and ZS. Data acquisition was performed by JM and OY. Software and data visualizations were created by JM. Writing of the original draft was performed by DV, JM, OY, and ZS. Editing and review of the manuscript was performed by DV, JM, HY, OY, and ZS.

### Conflict of Interest

The authors declare that the research was conducted in the absence of any commercial or financial relationships that could be construed as a potential conflict of interest.

## Supplementary Material

Software is available for download at: https://www.github.com/fda/SampPick.

Supplementary Table 1 The weighted and scaled frequencies of HLA-DRB1 variants in the North American population.
