# Supplementary material for: SampPick: Selection of a Cohort of Subjects Matching a Population HLA Distribution
Source: Front Immunol. 2019 Dec 20;10:2894. doi: 10.3389/fimmu.2019.02894 (PMC6933600; doi:10.3389/fimmu.2019.02894)
Supplement: Supplementary Table 1 — The weighted and scaled frequencies of HLA-DRB1 variants in the North American population. [file Table_1.docx]

## Supplementary Table

| **DRB1 Allele** | **Frequency in Population** |
| --- | --- |
| DRB1*01:01 | 6.61E-02 |
| DRB1*01:02 | 2.11E-02 |
| DRB1*01:03 | 7.39E-03 |
| DRB1*01:04 | 1.96E-06 |
| DRB1*01:06 | 3.07E-06 |
| DRB1*01:07 | 1.11E-05 |
| DRB1*01:08 | 3.61E-07 |
| DRB1*01:09 | 2.34E-07 |
| DRB1*01:10 | 7.40E-07 |
| DRB1*01:11 | 7.12E-07 |
| DRB1*01:16 | 3.90E-07 |
| DRB1*01:17 | 2.55E-07 |
| DRB1*01:18 | 5.46E-07 |
| DRB1*01:19 | 2.62E-07 |
| DRB1*01:20 | 9.00E-07 |
| DRB1*01:21 | 7.80E-08 |
| DRB1*01:22 | 6.24E-07 |
| DRB1*01:23 | 5.33E-07 |
| DRB1*01:24 | 7.80E-08 |
| DRB1*01:26 | 7.80E-08 |
| DRB1*01:28 | 7.80E-08 |
| DRB1*01:30 | 7.80E-08 |
| DRB1*01:31 | 0.00E+00 |
| DRB1*03:01 | 9.63E-02 |
| DRB1*03:02 | 1.09E-02 |
| DRB1*03:03 | 6.91E-06 |
| DRB1*03:04 | 8.27E-05 |
| DRB1*03:05 | 2.49E-05 |
| DRB1*03:06 | 9.33E-06 |
| DRB1*03:07 | 1.52E-05 |
| DRB1*03:08 | 7.80E-07 |
| DRB1*03:09 | 0.00E+00 |
| DRB1*03:10 | 7.80E-08 |
| DRB1*03:11 | 6.95E-07 |
| DRB1*03:12 | 3.12E-07 |
| DRB1*03:13 | 5.00E-06 |
| DRB1*03:14 | 4.45E-07 |
| DRB1*03:15 | 2.57E-05 |
| DRB1*03:16 | 3.39E-06 |
| DRB1*03:17 | 9.07E-07 |
| DRB1*03:18 | 6.77E-07 |
| DRB1*03:19 | 1.01E-06 |
| DRB1*03:20 | 1.33E-07 |
| DRB1*03:22 | 2.34E-07 |
| DRB1*03:23 | 4.20E-06 |
| DRB1*03:24 | 1.01E-06 |
| DRB1*03:25 | 3.00E-07 |
| DRB1*03:26 | 0.00E+00 |
| DRB1*03:27 | 4.77E-07 |
| DRB1*03:28 | 1.53E-06 |
| DRB1*03:29 | 4.00E-07 |
| DRB1*03:30 | 7.80E-08 |
| DRB1*03:31 | 0.00E+00 |
| DRB1*03:32 | 7.80E-08 |
| DRB1*03:33 | 2.67E-08 |
| DRB1*03:34 | 2.34E-07 |
| DRB1*03:36 | 3.97E-07 |
| DRB1*03:37 | 9.11E-07 |
| DRB1*03:38 | 2.67E-07 |
| DRB1*03:39 | 2.89E-07 |
| DRB1*03:40 | 0.00E+00 |
| DRB1*03:41 | 0.00E+00 |
| DRB1*03:43 | 1.31E-07 |
| DRB1*03:44 | 5.33E-09 |
| DRB1*03:47 | 7.80E-08 |
| DRB1*03:48 | 1.35E-07 |
| DRB1*03:49 | 7.80E-08 |
| DRB1*03:51 | 5.30E-08 |
| DRB1*03:52 | 7.45E-08 |
| DRB1*03:54 | 0.00E+00 |
| DRB1*03:57 | 5.66E-08 |
| DRB1*03:60 | 7.80E-08 |
| DRB1*03:63 | 2.83E-08 |
| DRB1*03:64 | 0.00E+00 |
| DRB1*04:01 | 5.63E-02 |
| DRB1*04:02 | 1.04E-02 |
| DRB1*04:03 | 1.15E-02 |
| DRB1*04:04 | 3.17E-02 |
| DRB1*04:05 | 1.36E-02 |
| DRB1*04:06 | 1.36E-03 |
| DRB1*04:07 | 1.90E-02 |
| DRB1*04:08 | 2.88E-03 |
| DRB1*04:09 | 5.35E-05 |
| DRB1*04:10 | 6.47E-04 |
| DRB1*04:11 | 3.83E-03 |
| DRB1*04:13 | 1.67E-05 |
| DRB1*04:14 | 1.33E-05 |
| DRB1*04:15 | 6.78E-06 |
| DRB1*04:16 | 2.79E-06 |
| DRB1*04:17 | 3.15E-05 |
| DRB1*04:18 | 1.61E-05 |
| DRB1*04:19 | 3.05E-06 |
| DRB1*04:21 | 7.80E-08 |
| DRB1*04:22 | 6.12E-07 |
| DRB1*04:23 | 1.93E-06 |
| DRB1*04:24 | 2.02E-07 |
| DRB1*04:25 | 4.42E-06 |
| DRB1*04:26 | 5.53E-06 |
| DRB1*04:27 | 1.06E-06 |
| DRB1*04:31 | 4.89E-07 |
| DRB1*04:32 | 1.37E-06 |
| DRB1*04:33 | 9.93E-07 |
| DRB1*04:34 | 6.24E-07 |
| DRB1*04:35 | 6.07E-06 |
| DRB1*04:36 | 3.43E-07 |
| DRB1*04:37 | 4.89E-07 |
| DRB1*04:38 | 5.17E-06 |
| DRB1*04:39 | 1.25E-06 |
| DRB1*04:40 | 4.42E-06 |
| DRB1*04:41 | 2.90E-06 |
| DRB1*04:42 | 7.80E-08 |
| DRB1*04:43 | 3.61E-07 |
| DRB1*04:44 | 3.65E-07 |
| DRB1*04:48 | 8.11E-08 |
| DRB1*04:50 | 3.74E-06 |
| DRB1*04:51 | 2.65E-07 |
| DRB1*04:54 | 2.11E-06 |
| DRB1*04:55 | 5.30E-08 |
| DRB1*04:56 | 3.90E-07 |
| DRB1*04:59 | 5.46E-07 |
| DRB1*04:60 | 2.84E-07 |
| DRB1*04:62 | 1.59E-07 |
| DRB1*04:67 | 0.00E+00 |
| DRB1*04:68 | 1.25E-06 |
| DRB1*04:69 | 5.30E-08 |
| DRB1*04:70 | 4.68E-07 |
| DRB1*04:71 | 5.46E-07 |
| DRB1*04:72 | 1.25E-06 |
| DRB1*04:74 | 5.30E-08 |
| DRB1*04:76 | 1.56E-07 |
| DRB1*04:77 | 4.68E-08 |
| DRB1*04:79 | 2.55E-07 |
| DRB1*04:83 | 3.12E-08 |
| DRB1*04:84 | 5.30E-08 |
| DRB1*04:86 | 0.00E+00 |
| DRB1*04:88 | 3.40E-07 |
| DRB1*04:89 | 5.30E-08 |
| DRB1*04:95 | 1.59E-07 |
| DRB1*07:01 | 1.20E-01 |
| DRB1*07:03 | 1.57E-05 |
| DRB1*07:04 | 7.80E-08 |
| DRB1*07:05 | 3.12E-05 |
| DRB1*07:07 | 6.21E-06 |
| DRB1*07:08 | 5.56E-07 |
| DRB1*07:09 | 1.71E-07 |
| DRB1*07:10 | 6.40E-07 |
| DRB1*07:11 | 5.39E-06 |
| DRB1*07:12 | 7.80E-08 |
| DRB1*07:13 | 4.17E-07 |
| DRB1*07:14 | 3.12E-07 |
| DRB1*07:15 | 3.83E-07 |
| DRB1*07:16 | 9.77E-07 |
| DRB1*07:17 | 0.00E+00 |
| DRB1*07:20 | 7.80E-08 |
| DRB1*07:21 | 7.80E-08 |
| DRB1*08:01 | 1.72E-02 |
| DRB1*08:02 | 1.39E-02 |
| DRB1*08:03 | 4.36E-03 |
| DRB1*08:04 | 1.10E-02 |
| DRB1*08:05 | 1.17E-05 |
| DRB1*08:06 | 9.86E-04 |
| DRB1*08:07 | 1.06E-04 |
| DRB1*08:08 | 5.26E-06 |
| DRB1*08:09 | 7.19E-05 |
| DRB1*08:10 | 8.97E-05 |
| DRB1*08:11 | 2.28E-04 |
| DRB1*08:12 | 5.02E-07 |
| DRB1*08:13 | 9.81E-06 |
| DRB1*08:14 | 9.54E-07 |
| DRB1*08:16 | 2.20E-07 |
| DRB1*08:17 | 7.77E-06 |
| DRB1*08:18 | 9.76E-06 |
| DRB1*08:19 | 1.82E-06 |
| DRB1*08:20 | 1.40E-06 |
| DRB1*08:21 | 0.00E+00 |
| DRB1*08:22 | 3.12E-07 |
| DRB1*08:23 | 5.30E-08 |
| DRB1*08:24 | 5.67E-07 |
| DRB1*08:25 | 8.00E-08 |
| DRB1*08:26 | 1.95E-06 |
| DRB1*08:27 | 5.30E-08 |
| DRB1*08:28 | 3.90E-07 |
| DRB1*08:30 | 6.72E-07 |
| DRB1*08:32 | 1.59E-07 |
| DRB1*08:34 | 4.71E-07 |
| DRB1*08:35 | 1.06E-07 |
| DRB1*08:39 | 0.00E+00 |
| DRB1*08:41 | 0.00E+00 |
| DRB1*08:42 | 1.28E-07 |
| DRB1*09:01 | 1.71E-02 |
| DRB1*09:02 | 1.05E-06 |
| DRB1*09:04 | 7.42E-07 |
| DRB1*09:06 | 4.86E-06 |
| DRB1*09:09 | 5.30E-08 |
| DRB1*10:01 | 1.36E-02 |
| DRB1*10:02 | 6.29E-07 |
| DRB1*10:03 | 2.84E-07 |
| DRB1*11:01 | 6.14E-02 |
| DRB1*11:02 | 9.27E-03 |
| DRB1*11:03 | 5.04E-03 |
| DRB1*11:04 | 2.94E-02 |
| DRB1*11:05 | 8.22E-06 |
| DRB1*11:06 | 1.56E-04 |
| DRB1*11:07 | 7.07E-06 |
| DRB1*11:08 | 4.49E-05 |
| DRB1*11:09 | 1.55E-05 |
| DRB1*11:10 | 1.78E-04 |
| DRB1*11:102 | 1.56E-07 |
| DRB1*11:103 | 7.80E-08 |
| DRB1*11:104 | 6.70E-08 |
| DRB1*11:108 | 6.23E-08 |
| DRB1*11:11 | 8.63E-05 |
| DRB1*11:12 | 4.45E-05 |
| DRB1*11:13 | 4.53E-05 |
| DRB1*11:14 | 7.99E-05 |
| DRB1*11:15 | 6.86E-05 |
| DRB1*11:16 | 6.07E-06 |
| DRB1*11:17 | 4.98E-05 |
| DRB1*11:18 | 1.09E-05 |
| DRB1*11:19 | 2.35E-05 |
| DRB1*11:20 | 1.80E-06 |
| DRB1*11:21 | 7.80E-07 |
| DRB1*11:22 | 1.01E-07 |
| DRB1*11:23 | 6.90E-07 |
| DRB1*11:24 | 1.02E-05 |
| DRB1*11:25 | 2.23E-06 |
| DRB1*11:26 | 7.95E-08 |
| DRB1*11:27 | 1.30E-05 |
| DRB1*11:28 | 2.10E-05 |
| DRB1*11:29 | 8.85E-06 |
| DRB1*11:30 | 1.15E-07 |
| DRB1*11:31 | 2.33E-08 |
| DRB1*11:32 | 2.32E-06 |
| DRB1*11:33 | 1.49E-06 |
| DRB1*11:34 | 9.67E-06 |
| DRB1*11:35 | 2.92E-06 |
| DRB1*11:36 | 1.13E-05 |
| DRB1*11:37 | 5.31E-06 |
| DRB1*11:38 | 1.56E-07 |
| DRB1*11:39 | 6.20E-06 |
| DRB1*11:40 | 7.51E-07 |
| DRB1*11:41 | 1.50E-06 |
| DRB1*11:42 | 1.80E-06 |
| DRB1*11:43 | 9.82E-06 |
| DRB1*11:45 | 3.32E-06 |
| DRB1*11:46 | 1.56E-07 |
| DRB1*11:47 | 1.92E-05 |
| DRB1*11:48 | 2.13E-06 |
| DRB1*11:49 | 6.56E-07 |
| DRB1*11:51 | 5.10E-07 |
| DRB1*11:52 | 5.30E-08 |
| DRB1*11:53 | 2.34E-07 |
| DRB1*11:54 | 6.08E-07 |
| DRB1*11:56 | 1.13E-06 |
| DRB1*11:57 | 1.59E-07 |
| DRB1*11:58 | 9.85E-07 |
| DRB1*11:60 | 2.06E-07 |
| DRB1*11:62 | 7.80E-08 |
| DRB1*11:65 | 5.35E-06 |
| DRB1*11:66 | 4.49E-07 |
| DRB1*11:68 | 5.30E-08 |
| DRB1*11:69 | 7.80E-07 |
| DRB1*11:72 | 1.33E-07 |
| DRB1*11:73 | 1.28E-07 |
| DRB1*11:75 | 5.30E-08 |
| DRB1*11:76 | 9.36E-07 |
| DRB1*11:83 | 1.81E-07 |
| DRB1*11:84 | 1.02E-06 |
| DRB1*11:85 | 7.80E-08 |
| DRB1*11:86 | 1.33E-07 |
| DRB1*11:87 | 1.28E-07 |
| DRB1*11:88 | 7.80E-08 |
| DRB1*11:89 | 7.80E-08 |
| DRB1*11:91 | 7.80E-08 |
| DRB1*11:92 | 5.30E-08 |
| DRB1*11:93 | 1.28E-07 |
| DRB1*11:95 | 2.93E-07 |
| DRB1*11:98 | 0.00E+00 |
| DRB1*12:01 | 1.92E-02 |
| DRB1*12:02 | 5.83E-03 |
| DRB1*12:03 | 3.75E-05 |
| DRB1*12:04 | 6.64E-06 |
| DRB1*12:05 | 2.30E-06 |
| DRB1*12:08 | 1.11E-06 |
| DRB1*12:09 | 7.80E-08 |
| DRB1*12:11 | 1.01E-06 |
| DRB1*12:12 | 5.30E-08 |
| DRB1*12:14 | 4.08E-08 |
| DRB1*12:15 | 4.08E-08 |
| DRB1*12:19 | 2.65E-07 |
| DRB1*12:20 | 1.06E-07 |
| DRB1*12:24 | 7.80E-08 |
| DRB1*13:01 | 5.59E-02 |
| DRB1*13:02 | 4.84E-02 |
| DRB1*13:03 | 1.44E-02 |
| DRB1*13:04 | 2.66E-03 |
| DRB1*13:05 | 2.50E-03 |
| DRB1*13:06 | 2.73E-05 |
| DRB1*13:07 | 1.50E-05 |
| DRB1*13:08 | 5.82E-06 |
| DRB1*13:09 | 5.99E-06 |
| DRB1*13:10 | 3.26E-05 |
| DRB1*13:102 | 5.86E-08 |
| DRB1*13:104 | 1.02E-07 |
| DRB1*13:106 | 2.83E-08 |
| DRB1*13:107 | 9.93E-08 |
| DRB1*13:11 | 2.54E-05 |
| DRB1*13:111 | 1.28E-07 |
| DRB1*13:12 | 2.01E-04 |
| DRB1*13:13 | 1.69E-06 |
| DRB1*13:14 | 2.31E-05 |
| DRB1*13:15 | 2.51E-05 |
| DRB1*13:16 | 5.13E-05 |
| DRB1*13:17 | 9.34E-07 |
| DRB1*13:18 | 1.10E-05 |
| DRB1*13:19 | 1.56E-05 |
| DRB1*13:20 | 1.15E-05 |
| DRB1*13:21 | 1.79E-05 |
| DRB1*13:22 | 1.61E-05 |
| DRB1*13:23 | 2.53E-06 |
| DRB1*13:24 | 4.49E-06 |
| DRB1*13:25 | 1.15E-06 |
| DRB1*13:26 | 1.66E-06 |
| DRB1*13:27 | 5.35E-06 |
| DRB1*13:28 | 1.53E-06 |
| DRB1*13:29 | 3.42E-06 |
| DRB1*13:30 | 2.12E-07 |
| DRB1*13:31 | 4.16E-05 |
| DRB1*13:32 | 2.53E-07 |
| DRB1*13:33 | 7.02E-07 |
| DRB1*13:34 | 2.68E-07 |
| DRB1*13:36 | 1.12E-05 |
| DRB1*13:37 | 8.50E-06 |
| DRB1*13:38 | 5.51E-06 |
| DRB1*13:39 | 1.39E-06 |
| DRB1*13:40 | 9.62E-06 |
| DRB1*13:41 | 2.31E-06 |
| DRB1*13:42 | 2.62E-06 |
| DRB1*13:44 | 2.34E-07 |
| DRB1*13:45 | 2.11E-07 |
| DRB1*13:47 | 5.30E-08 |
| DRB1*13:48 | 3.12E-07 |
| DRB1*13:49 | 8.19E-06 |
| DRB1*13:50 | 6.35E-07 |
| DRB1*13:51 | 1.56E-07 |
| DRB1*13:52 | 1.02E-06 |
| DRB1*13:53 | 5.74E-07 |
| DRB1*13:54 | 7.02E-07 |
| DRB1*13:55 | 3.44E-07 |
| DRB1*13:56 | 3.91E-06 |
| DRB1*13:59 | 1.75E-05 |
| DRB1*13:60 | 4.11E-07 |
| DRB1*13:61 | 1.59E-06 |
| DRB1*13:62 | 1.56E-07 |
| DRB1*13:63 | 3.65E-07 |
| DRB1*13:65 | 3.90E-07 |
| DRB1*13:66 | 3.90E-07 |
| DRB1*13:67 | 2.34E-07 |
| DRB1*13:68 | 1.62E-07 |
| DRB1*13:69 | 4.78E-07 |
| DRB1*13:70 | 8.93E-08 |
| DRB1*13:71 | 0.00E+00 |
| DRB1*13:76 | 4.26E-08 |
| DRB1*13:77 | 2.84E-07 |
| DRB1*13:78 | 0.00E+00 |
| DRB1*13:79 | 1.88E-06 |
| DRB1*13:80 | 2.11E-07 |
| DRB1*13:81 | 1.93E-06 |
| DRB1*13:82 | 2.10E-06 |
| DRB1*13:83 | 1.99E-07 |
| DRB1*13:84 | 8.93E-08 |
| DRB1*13:85 | 1.33E-07 |
| DRB1*13:86 | 1.56E-07 |
| DRB1*13:87 | 1.13E-08 |
| DRB1*13:88 | 4.64E-07 |
| DRB1*13:90 | 9.70E-08 |
| DRB1*13:91 | 8.36E-08 |
| DRB1*13:92 | 7.80E-08 |
| DRB1*13:93 | 8.45E-07 |
| DRB1*13:94 | 7.80E-08 |
| DRB1*13:95 | 1.28E-07 |
| DRB1*13:96 | 7.80E-08 |
| DRB1*13:97 | 1.62E-07 |
| DRB1*13:98 | 5.30E-08 |
| DRB1*14:01 | 2.42E-02 |
| DRB1*14:02 | 4.86E-03 |
| DRB1*14:03 | 2.26E-04 |
| DRB1*14:04 | 2.71E-03 |
| DRB1*14:05 | 7.96E-04 |
| DRB1*14:06 | 5.39E-03 |
| DRB1*14:07 | 4.51E-04 |
| DRB1*14:08 | 2.95E-05 |
| DRB1*14:09 | 1.75E-06 |
| DRB1*14:10 | 7.50E-06 |
| DRB1*14:100 | 0.00E+00 |
| DRB1*14:101 | 2.51E-05 |
| DRB1*14:102 | 3.18E-08 |
| DRB1*14:103 | 0.00E+00 |
| DRB1*14:104 | 0.00E+00 |
| DRB1*14:105 | 0.00E+00 |
| DRB1*14:106 | 6.38E-07 |
| DRB1*14:107 | 7.80E-08 |
| DRB1*14:108 | 0.00E+00 |
| DRB1*14:109 | 1.28E-07 |
| DRB1*14:11 | 1.60E-05 |
| DRB1*14:110 | 0.00E+00 |
| DRB1*14:111 | 0.00E+00 |
| DRB1*14:112 | 0.00E+00 |
| DRB1*14:113 | 0.00E+00 |
| DRB1*14:12 | 1.24E-05 |
| DRB1*14:13 | 3.95E-06 |
| DRB1*14:15 | 3.25E-05 |
| DRB1*14:16 | 1.30E-05 |
| DRB1*14:17 | 4.12E-05 |
| DRB1*14:18 | 2.46E-05 |
| DRB1*14:19 | 8.60E-06 |
| DRB1*14:20 | 5.53E-06 |
| DRB1*14:21 | 3.32E-06 |
| DRB1*14:22 | 2.97E-06 |
| DRB1*14:23 | 1.22E-06 |
| DRB1*14:24 | 6.39E-05 |
| DRB1*14:25 | 4.07E-06 |
| DRB1*14:27 | 1.17E-06 |
| DRB1*14:28 | 1.48E-06 |
| DRB1*14:29 | 1.10E-06 |
| DRB1*14:30 | 0.00E+00 |
| DRB1*14:32 | 3.90E-07 |
| DRB1*14:33 | 5.25E-06 |
| DRB1*14:34 | 0.00E+00 |
| DRB1*14:35 | 5.76E-07 |
| DRB1*14:36 | 2.46E-07 |
| DRB1*14:38 | 1.81E-07 |
| DRB1*14:39 | 5.30E-08 |
| DRB1*14:41 | 3.42E-06 |
| DRB1*14:42 | 1.07E-06 |
| DRB1*14:44 | 1.04E-06 |
| DRB1*14:45 | 0.00E+00 |
| DRB1*14:46 | 1.28E-07 |
| DRB1*14:47 | 1.19E-06 |
| DRB1*14:48 | 1.46E-05 |
| DRB1*14:50 | 7.40E-07 |
| DRB1*14:52 | 1.51E-06 |
| DRB1*14:53 | 0.00E+00 |
| DRB1*14:55 | 7.80E-08 |
| DRB1*14:58 | 2.34E-07 |
| DRB1*14:60 | 7.80E-08 |
| DRB1*14:61 | 7.42E-07 |
| DRB1*14:62 | 0.00E+00 |
| DRB1*14:67 | 2.97E-08 |
| DRB1*14:68 | 2.65E-07 |
| DRB1*14:69 | 2.65E-07 |
| DRB1*14:70 | 1.56E-06 |
| DRB1*14:71 | 1.59E-07 |
| DRB1*14:72 | 2.06E-07 |
| DRB1*14:73 | 2.12E-07 |
| DRB1*14:75 | 2.34E-07 |
| DRB1*14:77 | 2.12E-08 |
| DRB1*14:79 | 7.80E-08 |
| DRB1*14:81 | 1.28E-07 |
| DRB1*14:83 | 1.28E-07 |
| DRB1*14:93 | 5.30E-08 |
| DRB1*14:94 | 6.47E-07 |
| DRB1*14:95 | 7.80E-08 |
| DRB1*14:96 | 5.30E-08 |
| DRB1*14:97 | 1.31E-07 |
| DRB1*14:99 | 7.80E-08 |
| DRB1*15:01 | 1.00E-01 |
| DRB1*15:02 | 1.39E-02 |
| DRB1*15:03 | 1.84E-02 |
| DRB1*15:04 | 6.98E-05 |
| DRB1*15:05 | 9.85E-07 |
| DRB1*15:06 | 2.86E-04 |
| DRB1*15:07 | 6.64E-06 |
| DRB1*15:08 | 1.94E-06 |
| DRB1*15:09 | 2.11E-07 |
| DRB1*15:10 | 4.02E-06 |
| DRB1*15:11 | 1.81E-06 |
| DRB1*15:13 | 2.42E-05 |
| DRB1*15:14 | 4.67E-06 |
| DRB1*15:15 | 3.12E-07 |
| DRB1*15:17 | 1.28E-07 |
| DRB1*15:18 | 4.96E-06 |
| DRB1*15:20 | 1.06E-06 |
| DRB1*15:21 | 0.00E+00 |
| DRB1*15:22 | 2.25E-06 |
| DRB1*15:23 | 4.00E-06 |
| DRB1*15:24 | 1.72E-06 |
| DRB1*15:25 | 7.80E-08 |
| DRB1*15:26 | 5.30E-08 |
| DRB1*15:27 | 1.56E-07 |
| DRB1*15:31 | 1.59E-07 |
| DRB1*15:32 | 1.28E-07 |
| DRB1*15:33 | 7.80E-08 |
| DRB1*15:37 | 7.80E-08 |
| DRB1*15:38 | 5.83E-07 |
| DRB1*15:41 | 2.21E-07 |
| DRB1*15:42 | 7.80E-08 |
| DRB1*15:44 | 5.30E-08 |
| DRB1*15:48 | 0.00E+00 |
| DRB1*15:50 | 0.00E+00 |
| DRB1*15:52 | 2.01E-07 |
| DRB1*16:01 | 1.28E-02 |
| DRB1*16:02 | 8.19E-03 |
| DRB1*16:03 | 0.00E+00 |
| DRB1*16:04 | 4.61E-06 |
| DRB1*16:05 | 3.01E-05 |
| DRB1*16:07 | 8.89E-06 |
| DRB1*16:08 | 9.85E-07 |
| DRB1*16:09 | 2.87E-07 |
| DRB1*16:10 | 2.02E-06 |
| DRB1*16:12 | 1.79E-06 |
| DRB1*16:14 | 4.00E-07 |
| DRB1*16:15 | 6.38E-07 |
| DRB1*16:18 | 5.30E-08 |
